# Bamboo: an overview on its genetic diversity and characterization

**DOI:** 10.1007/s13205-014-0201-5

**Published:** 2014-03-01

**Authors:** Lucina Yeasmin, Md. Nasim Ali, Saikat Gantait, Somsubhra Chakraborty

**Affiliations:** 1 Department of Agricultural Biotechnology, Faculty Centre for Integrated Rural Development and Management, School of Agriculture and Rural Development, Ramakrishna Mission Vivekananda University, Ramakrishna Mission Ashrama, Narendrapur, Kolkata, 700103 India; 2 Department of Crop Science, Faculty of Agriculture, Universiti Putra Malaysia, 43400 Serdang, Selangor Malaysia; 3 Department of Biotechnology, Instrumentation and Environmental Science, Bidhan Chandra Krishi Viswavidyalaya, Mohanpur, WB 741252 India

**Keywords:** *Bambusa*, Biodiversity, *Dendrocalamus*, Molecular marker

## Abstract

Genetic diversity represents the heritable variation both within and among populations of organisms, and in the context of this paper, among bamboo species. Bamboo is an economically important member of the grass family Poaceae, under the subfamily Bambusoideae. India has the second largest bamboo reserve in Asia after China. It is commonly known as “poor man’s timber”, keeping in mind the variety of its end use from cradle to coffin. There is a wide genetic diversity of bamboo around the globe and this pool of genetic variation serves as the base for selection as well as for plant improvement. Thus, the identification, characterization and documentation of genetic diversity of bamboo are essential for this purpose. During recent years, multiple endeavors have been undertaken for characterization of bamboo species with the aid of molecular markers for sustainable utilization of genetic diversity, its conservation and future studies. Genetic diversity assessments among the identified bamboo species, carried out based on the DNA fingerprinting profiles, either independently or in combination with morphological traits by several researchers, are documented in the present review. This review will pave the way to prepare the database of prevalent bamboo species based on their molecular characterization.

## Introduction

### Bamboo: Taxonomy

Bamboo, the fastest growing perennial, evergreen, arborescent plant is a member of the grass family (i.e., Poaceae) and constitutes a single subfamily Bambusoideae (Kigomo 1988). The Bambusoideae subfamily includes both herbaceous bamboo or Olyreae tribe and woody bamboos or Bambuseae tribe (Ramanayake et al. 2007). The Bambuseae tribe differs from the Olyreae on the basis of the presence of abaxial ligule (Zhang and Clark 2000; Grass Phylogeny Working Group 2001). The most recent classification systems have placed 67 genera of woody bamboos under nine subtribes, mainly depending on various floral characters (Dransfield and Widjaja 1995; Li 1997).

### Area and distribution

**Fig. 1:**
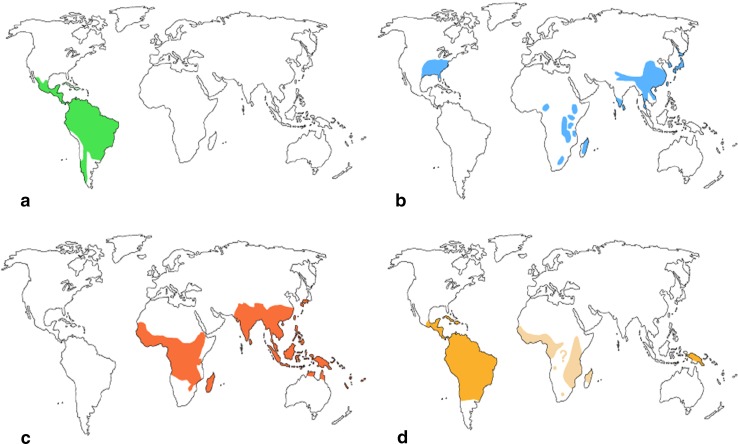
Worldwide distribution of bamboo. **a** Neotropical woody bamboos, **b** north temperate woody bamboos, **c** paleotropical woody bamboos, and **d** herbaceous bamboos (Source: http://www.eeob.iastate.edu/bamboo/maps.html)

The distribution of bamboos on planet earth extends from 51°N latitude in Japan (Island of Sakhalin) to 47°S latitude in South Argentina. A total number of 1,400 bamboo species are distributed worldwide. The bamboo can grow in an altitudinal range which extends from just above the sea level up to 4,000 m (Behari 2006). About 14 million hectares of the earth surface is covered by bamboos with 80 percent in Asia (Tewari 1992). The major species richness is found in Asia-pacific followed by South America, whereas the least number of species is found in Africa (Bystriakova et al. 2003). It has been reported that Europe has no native bamboo species (Liese and Hamburg 1987). According to FAO, total area under bamboo cultivation is 11,361 ha as in 2005, of which 1,754 ha is under private ownership. Herbaceous bamboo constitutes about 110 species which are mainly concentrated in the Neotropics of Brazil, Paraguay, Mexico and West Indies. The natural bamboo forest covers approximately 600,000 ha area across Brazil, Peru and Bolivia, which is known as “Tabocais” in Brazil and “Pacales” in Peru (Filgueiras and Goncalves 2004 cited in Das et al. 2008). The Bambuseae tribe includes about 1,290 species worldwide and constitutes three major groups (Das et al. 2008). The Paleotropical woody bamboo is distributed in the tropical and subtropical regions of Africa, Madagascar, Sri Lanka, India, Southern Japan, Southern China and Oceania. The Neotropical woody bamboos are distributed in Southern Mexico, Argentina, Chile and West Indies. The north temperate woody bamboos are found in the North Temperate Zone and a small amount at a higher elevation of Madagascar, Africa, India and Sri Lanka (http://www.eeob.iastate.edu/bamboo/maps.html) (Fig. 1). Bamboo can thrive in hot, humid rainforests to cold resilient forests. It can tolerate as well as can grow in extreme temperature of about −20 °C. It also can tolerate excessive precipitation ranging from 32 to 50 inch. annual rainfall (Goyal et al. 2012).

### Bamboo in India

**Fig. 2:**
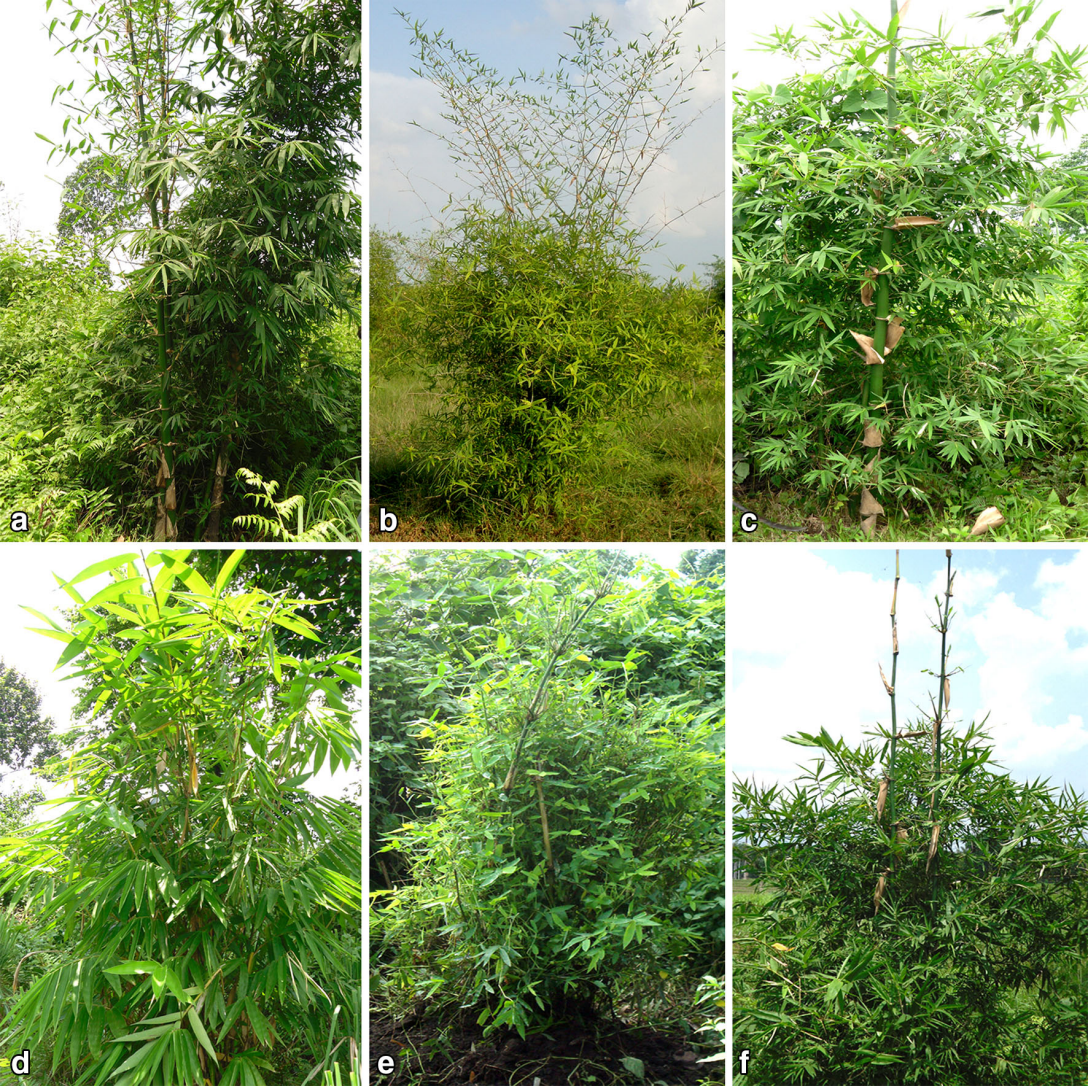
*Dendrocalamus* and *Bambusa*, the two most predominant bamboo species distributed in subtropical, tropical moist and tropical dry agroclimatic zones of India. **a***Bambusa balcooa*, **b***Bambusa bambos*, **c***Bambusa tulda*, **d***Dendrocalamus asper*, **e***Dendrocalamus hamiltonii*, **f***Dendrocalamus strictus* (Source: Authors)

India is the second richest country in bamboo genetic resources following China, ranking first in this aspect (Bystriakova et al. 2003). Several reports have been found regarding the species richness of bamboo in India. Bahadur and Jain (1983) reported about 113 bamboo species, whereas reports on the number of species varies from 102 (Ohrnberger 2002) to 136 (Sharma 1980). In India, 9.57 million ha which is about 12.8 % of the total forest area of the country is covered by bamboo plantation (Sharma 1980). As opined by many scientists, the distribution of bamboo is greatly influenced by human interventions (Boontawee 1988). However, Gamble (1896) has earlier reported that the distribution of bamboo in India is related to rainfall. Varmah and Bahadur (1980) in another report have associated the preferential distribution of different bamboo species with different agroclimatic zones of India. The alpine region comprises *Arundinaria* and *Thamnocalamus*, whereas, these two genera grow in the temperate region along with *Phyllostachys*. *Arundinaria*, *Bambusa* and *Dendrocalamus* grow in the subtropical region, the tropical moist region allows to grow *Bambusa*, *Dendrocalamus*, *Melocanna*, *Ochlandra* and *Oxytenanthera*; on the other hand, in the dry tropical region *Dendrocalamus* and *Bambusa* is predominant (Ahmed 1996) (Fig. 2).

### Chromosome and genetic status

Bamboo under the subfamily Bambusoideae is the giant member of grass family (Kigomo 1988). The basic chromosome number of most woody bamboo is 12 (*x* = 12), whereas in herbaceous bamboo it is 11 (*x* = 11) (Grass Phylogeny Working Group 2001). Two different polyploidy groups are present in woody bamboo. The tropical woody bamboo is hexaploid (*2n* = *6x* = 72) and the temperate woody bamboo is tetraploid (*2n* = *4x* = 48) (Clark et al. 1995). After employing an exhaustive chromosome analysis on 185 species from 33 genera and 6 subtribes, Ruiyang (2003) has reported the variation in the chromosome numbers for some species of *Bambusa* and *Dendrocalamus*. The genomic DNA content of tropical woody bamboo is larger than that of the temperate woody bamboo as estimated by flow cytometric analysis (Gielis et al. 1997). The recent flow cytometric analysis of tetraploid bamboo *Phyllostachys heterocycla* var. *pubescens* has estimated its genome size to be 2.075 Gb (Peng et al. 2013). The number of ESTs of bamboo deposited till January 2009 is 3,087 (Peng et al. 2010) and total number of nucleotide sequences deposited till November 2009 is 17,789 (which is <0.1 % of the total sequences from grass family). As reported by Peng et al. (2013), the moso bamboo genome contains 43.9 % GC and 59.0 % transposable elements. In the same article, they have reported 31,987 protein coding genes and the average length of protein coding gene is 3,350 bp.

### Bamboo and its usage: the poor man’s timber

Bamboo has age-old connection with the material needs of rural people (Mukherjee et al. 2010). Porter-field (1933) suggested that “bamboo is one of those providential developments in nature which, like the horse, the cow, wheat and cotton, have been indirectly responsible for man’s own evolution”. Bamboo plays manifold role in day-to-day rural life or broadly speaking human life. It plays a crucial role in cultural, artistic, industrial, agricultural, construction and household needs of human beings (McNeely 1995). The utility of bamboo shoots and leaves vary from pickle preparation (Khatta and Katoch 1983) to preparation of traditional medicine. Bamboo has medicinal values too. It has multiple wide uses in ayurveda. Solvent extraction of *P. pubescens* and *P. bambusoideae* showed strong antioxidant activity (Mu et al. 2004). The mature bamboo leaves contain phenolic acids and root contains cyanogenic glycosides (Das et al. 2012). From the adult bamboo culms, high-quality charcoal is produced (Park and Kwon 1998). In some parts of South East Asia, life starts with a knife made of bamboo as the umbilical cord of a new born baby is cut by it (McNeely 1995), and also it is utilized for the procedure of circumcision of a male child (Skeat 1900). Bamboo plays a significant role in paper and pulp industry. As reported by Sharma et al., the national demand of bamboo was 5 million tonnes by 1987 out of which 3.5 million tonnes were required for paper and pulp industry. *B. balcooa* is generally chosen for construction purposes and fiber-based mat board and panel manufacture (Ganapathy 1997), but for its mechanical strength it is also utilized to produce quality paper pulp (Das et al. 2005). Bamboo is considered as “green gold” keeping in mind its economic importance and multiple end uses in human life (Bhattacharya et al. 2009). Bamboo is widely utilized for making various musical instruments, e.g., flutes are made from hollow bamboo (Kurz 1876). Bamboo is widely utilized for construction purposes. It has manifold application in construction of house such as making pillars, floors, doors and windows, room separator, rafters etc. (Das et al. 2008). It is also utilized for making guard wall of water bodies and river bank. Bamboo is an efficient agent for preventing soil erosion and conserving soil moisture (Christanty et al. 1996, 1997; Mailly et al. 1997; Kleinhenz and Midmore 2001).

### Bamboo and biodiversity

In addition to the countless direct human uses, bamboo plays an imperative role in many other ways; as per Kratter’s survey, 25 out of 440 bird species living in the Amazon forest are confined to bamboo thickets. Elephants (*Elephas maximus*), wild cattle (*Bos gaurus* and *B. javanicus)* and various species of deer (*Cervidae*) and primates (including macaques Macaca and leaf monkeys *Presbytis),* pigs (Suidae), rats and mice (Muridae), porcupines (Hystricidae) and squirrels (Sciuridae) are subsidiary feeders on Southeast Asian bamboos. More than 15 Asian bird species nests exclusively in bamboo; many of these are rare and threatened using bamboo as the significant proportion of their habitat (Bird Life International 2000). The world’s second smallest bat (*Tylonycteris pachypus*, 3.5 cm) nests between nodes of mature bamboo (*Gigantochloa scortechinii*), which it enters through holes created by beetles. The Asian giant panda (*Ailuropoda melanoleuca*), red panda (*Ailurus fulgens*) and the Himalayan black bear (*Selenarctos thibetanus*) are heavily dependent on bamboo for their feed (Bystriakova et al. 2003). The Red Panda (*A. fulgens*) is recently listed as endangered in the Red Data Book of IUCN. Red Panda mainly lives on bamboo leaves and the destruction of bamboo forest is one of the main reasons for its extinction from the wild (Red panda network, www.redpandanetwork.org). Leaves of *Sasa senanesis*, *S. kurilensis* and *S. nipponica* constitute a major part of the winter diet for Hokkaido voles (*Clethrionomys rufocanus*), it is when most other plants wither.

### Importance of characterization

Overexploitation and genetic erosion of bamboo species have made it necessary not only for the collection and conservation of its germplasms (Thomas et al. 1988; Loh et al. 2000) but also to classify and characterize them (Bahadur 1979; Soderstorm and Calderon 1979; Rao and Rao 2005). Characterization of germplasm is an important link between the conservation and utilization of germplasms (Stapleton and Rao 1995; Nayak et al. 2003). To maintain the germplasms and conservation of biodiversity, the investigation of bamboo resources and even study of their local distribution is indispensable (Goyal et al. 2012); which is recorded to be limited till date.

### Identification of bamboo

Identification and classification is necessary for collection and conservation of germplasms (Bahadur 1979; Soderstorm and Calderon 1979). Overexploitation and genetic erosion has posed a foremost need for conservation of bamboo germplasm (Thomas et al. 1988; Loh et al. 2000). In case of any plant, the identification keys are mostly based on floral characters. Depending on the flowering cycle, the bamboos are categorized into three major groups, viz. annual flowering bamboos *(Indocalamus wightianus*, *Ochlandra sp.)*, sporadic or irregular flowering bamboos (*Chimonobambusa sp., D. hamiltonii*) and gregarious flowering bamboos (*B. bambos, B. tulda, D. strictus, T. spathiflora*) (Das et al. 2008; Bhattacharya et al. 2006, 2009). In case of gregarious flowering, all the members of a common cohort (plants from seeds of common origin) go into reproductive phase simultaneously and subsequently die (Bhattacharya et al. 2009). Incidence of flowering of woody bamboo is uncertain (Ramanayake et al. 2007; Mukherjee et al. 2010). As reported earlier, the reproductive cycle of bamboo is too long, from 3 to 120 years (Janzen 1976), hence making the identification depending on reproductive structure difficult (Bhattacharya et al. 2006, 2009). Consequently, the focus on identification of bamboo has shifted from reproductive to vegetative characters (Bhattacharya et al. 2006; Sharma et al. 2008).

Classification of bamboo was traditionally based on morphological characters; however, recently several other useful taxonomic information such as biochemical, anatomical and molecular characters have also been explored (Stapleton 1997). Even though, characterization of bamboos has so far been done based on morphological characters, yet the classification is not reliable since these are often influenced by ecological factors. Das et al. (2007) in their work have shown that only vegetative characters are unable to distinguish closely related species. The clustering pattern they obtained using the key morphological descriptors was not fully in agreement with the classification pattern of Gamble (1896). The reliability of taxonomic groupings based only on the morphological characters has often been questioned due to the involvement of small number of genes for morphological traits that may not truly reflect the entire scenario of the genome (Brown-Guedira et al. 2000 cited in Das et al. 2007).

Nevertheless, DNA-based marker or molecular markers are not influenced by environment (Ram et al. 2008) and it is thus reliable for diversity analysis. Although application of molecular technique for diversity analysis in bamboo was limited till the beginning of twenty-first century (Loh et al. 2000). In recent years, the application of molecular technology for identification and characterization of bamboo species is predominant.

### Morphological traits: key to bamboo identification and characterization

As early as in the year of 1896, J.S. Gamble identified old world bamboos based on various vegetative and reproductive characters. Later, botanists discovered different characters of culm-sheath and other vegetative organs as potential descriptors. Chatterjee and Raizada (1963) prepared a key to identification for 22 bamboo taxa based on culm-sheath morphology. According to them, “The general appearance, size, texture and shape of the sheath and their blades afford good characters for distinguishing the different species”. Branching pattern is an important characteristic for identification of genus (Bennet and Gaur 1990). Bennet and Gaur (1990) further suggested the study of young vegetative shoots which sprout annually during rainy season revealing that they are of highly distinct character and hence can be utilized as identification of different species.

To assess the relationship and diversity due to ecological and geographical variation within the members of *Chusquea culeou* species complex, 7 vegetative characters and 14 reproductive or floral characters were studied by Triplett and Clark (2003). The Principle Component Analysis based on vegetative and reproductive characters showed that the variation in the characters is continuous and cannot be used to demarcate the species into a morphologically distinct group. Their study emphasized that additional studies are necessary to resolve the classification of *C. culeou* species complex. In the same year, Clark (2003) identified a new species *C. renvoizei* classified within *Chusquea* section *Swallenochloa* based on different morphological characters. The species is endemic to Bolivia. For identification of that particular species viz. *C. renvoizei*, 10 quantitative and qualitative morphological characters were assessed. A number of vegetative and foliage features distinguished the species from the other members of the Swallenochloa section under *Chusquea* species complex. Lately, Bhattacharya et al. (2006) have described 15 culm and 17 culm-sheath characteristics which they have studied for characterization of *B. tulda*, a sporadically flowering bamboo. They surveyed natural bamboo stands from 17 eco-geographical locations in different districts of West Bengal. Their study was in conformity with the prior taxonomic classification given by Gamble (1896), but a detailed description and illustrations are presented in their article. Phylogenetic relationships among 15 bamboo species were evaluated by Das et al. (2007) using 32 key quantitative and qualitative morphological characters (15 culm and 17 culm-sheath characters), which were previously utilized by Bhattacharya et al. (2006). The cluster pattern obtained from key morphological descriptors was not in conforming to classification of Gamble (1896). The comprehensive morphological characterization was done in a gregarious flowering bamboo species, *T. spathiflorus* (Trin.) Munro subsp. *Spathiflorus* (Bhattacharya et al. 2009). An assembly of 28 key vegetative and reproductive characters was studied by them. The vegetative and floral morphology described was in gross agreement with previous reports given by Naithani et al. (2003) and Clayton et al. (2013). Even though, characterization of bamboos has so far been done based on morphological characters, yet the classification is not reliable since these are often influenced by ecological factors.

### Limitation of morphological characters

The reproductive cycle of bamboo is too long, from 3 to 120 years (Janzen 1976). So characterization and identification using floral characters is difficult (Bhattacharya et al. 2006, 2009). In case of bamboo, the taxonomical classification is based on mainly vegetative characters (Ohrnberger 2002). The vegetative characters are influenced by environment and therefore, not reliable for taxonomic classification. Triplett and Clark’s (2003) study concluded that only morphological descriptors were unable to demarcate the species into morphologically distinct group. As reported by Das et al. (2007), only vegetative characters are unable to distinguish closely related species. The dendrogram pattern of 15 bamboo species is not in agreement with classification given by Gamble (1896). *B. striata* and *B. wamin* of *Bambusa* genus were separated from other species of *Bambusa*. *D. strictus* was grouped with *B. striata*, *B. wamin* and *B. atra*.

### Bamboo and molecular descriptors

Molecular methods have become an indispensable part of most of the genetic diversity assay and in the analyses of breeding system, bottlenecks and other key features influencing genetic diversity patterns. The studies may use Restriction Fragment Length Polymorphism (RFLP), Randomly Amplified Polymorphic DNA (RAPD), Amplified Fragment Length polymorphism (AFLP) or Simple Sequence Repeat (SSR). Nevertheless, it is important to understand that different markers have different properties and will reflect different aspects of genetic diversity (Karp and Edwards 1995). Molecular data can provide useful information to deal with various aspects of taxonomic classification of plants (Das et al. 2008). “Molecular DNA techniques allow researchers to identify genotypes at the taxonomic level, assess the relative diversity within and among the species and locate diverse accessions for breeding purposes” (Nayak et al. 2003). As reported by Loh et al. (2000), the application of molecular techniques for genetic diversity assessment of bamboo was limited till 2000. The study included RFLP in *Phyllostachys* by Friar and Kochert (1994), isozyme analysis of few selection from 5 genera of bamboo by Heng et al. (1996), chloroplast DNA phylogeny of Asian bamboos by Watanabe et al. (1994) and world bamboo by Kobayashi (1997), analysis of *rpl16* intron sequences in determining phylogenetic relationship within the genus *Chusquea* (Loh et al. 2000).

There are many molecular markers available such as hybridization-based marker or RFLP marker, polymerase chain reaction (PCR) based markers such as RAPD, AFLP, SSR, Inter Simple Sequence Repeats (ISSR), Single Nucleotide Polymorphism (SNP) markers etc. RFLP technique was applied to study the genetic variation and evolution of 20 species of *Phyllostachys* by Friar and Kochert (1994). However, RFLP technique requires fine quality DNA and it shows very low polymorphism in comparison to others. RAPD is a low-cost and rapid method and does not require any information regarding the genome of the plant, and it also has been widely used to determine the genetic diversity in several plants (Belaj et al. 2001; Deshwall et al. 2005). As it is a quick and sensitive method, RAPD can be effectively employed to distinguish useful polymorphism (Ko et al. 1998). It requires very small amount of genomic DNA and can produce very high level of polymorphism and can be effective for diversity analysis in plants (Williams et al. 1990). RAPD analysis has proved its significance for diversity study of field crops like rice (Qian et al. 2001; Rabbani et al. 2008; Pervaiz et al. 2010), many horticultural plants such as coffee (Orozco-Castillo et al. 1994), tea (Wachira et al. 1995), almond (Shiran et al. 2007), sesame (Akbar et al. 2011), turmeric (Singh et al. 2012). It has been employed for phylogenetic relationship study and characterization of bamboo by many recent workers (Nayak et al. 2003; Das et al. 2005; Bhattacharya et al. 2006; Ramanayake et al. 2007; Das et al. 2007; Bhattacharya et al. 2009).

A sum of 98 mapped SSR primers from rice and 20 EST-derived SSR primers from sugarcane was utilized for the evaluation of genetic diversity among 23 bamboo species (Sharma et al. 2008). The study showed that 44 rice SSR and 15 SSR of sugarcane primers produced repeatable amplification in at least one species of bamboo. A total number of 42 out of these 59 primers proved to be efficient for species identification. Two species-specific Sequence Characterized Amplified Region (SCAR) markers were developed by Das et al. (2005). They have developed Bb_836_ for *B. balcooa* and Bt_609_ for *B. tulda*. The species specificity was confirmed by southern hybridization, and validation was done using 80 accessions of *B. balcooa* and *B. tulda* each. Recent genomic studies in bamboo include the study by Peng et al. (2010) in *P. pubescence* var. *heterocycla*. They have reported 10,608 full-length cDNA sequences of bamboo. Approximately 38,000 Expressed Sequence Tags (ESTs) were generated in this study. In the next year, Zhang et al. (2011) reported full genome sequence of six woody bamboo chloroplast genome (cp DNA). A contemporary study conducted by Gui et al. (2010) reports, identification of syntenic genes between bamboo and other grasses, such as rice and sorghum. They found that the content of repetitive elements (36.2 %) in bamboo is similar to that of rice. It was reported that both rice and sorghum express high genomic synteny with bamboo thus suggesting that these could be utilized as model for decoding tropical bamboo genomes.

Recent study includes phylogenetic analysis of Bambusoideae subspecies (Sungkaew et al. 2009), genome-wide full-length cDNA sequencing (Peng et al. 2010), identification of syntenic genes between bamboo and other grasses (Gui et al. 2010), chloroplast genome sequencing (Zhang et al. 2011) and identification of genes involved in fiber development (Rai et al. 2011). For analysis of genetic diversity of 23 bamboo accessions, 59 SSR from rice and sugarcane were utilized (Sharma et al. 2008). Two species-specific SCAR markers were developed and validated for identification of *B. tulda* and *B. balcooa* (Das et al. 2005) and very recently the draft genome of moso bamboo was reported by Peng et al. (2013). Sixteen novel microsatellite markers were developed for *D. sinicus* by Dong et al. (2012) which will be useful for evaluation of genetic diversity of *D. sinicus*. Very recently, the complete genome sequence of moso bamboo (*P. pubescence* var. *heterocycla)* was reported by Peng et al. (2013). The 2.05 Gb assembly covered 95 % of the genomic region and gene prediction modeling identified 31,987 genes.

### RAPD and ISSR: two most potential markers for bamboo genetic diversity study

RAPD is an inexpensive, simple and rapid technology (Belaj et al. 2001; Deshwall et al. 2005) which has been employed in diversity analysis in plants since its development by Williams et al. (1990). It requires small amount of genomic DNA and can produce high level of polymorphism (Williams et al. 1990). As reported by Ko et al. (1998), while studying genetic relationship within *Viola sp.*, RAPD, being a quick and sensitive method, can be utilized to distinguish polymorphism. RAPD analysis has proved its significance for diversity analysis and identification of germplasms of several plants (Kapteyn and Simon 2002; Welsh and McClelland 1990). RAPD has several limitations including dominance, uncertain locus homology, and especially sensitivity to the reaction conditions as reported by Qian et al. (2001). According to them to solve some of these problems, Inter Simple Sequence Repeat (ISSR) markers can be put into effect.

ISSRs are the regions that lie within the microsatellite repeats (Joshi et al. 2000) and offer great potential to determine intra-genomic and inter-genomic diversity compared to other arbitrary primers, since they reveal variation within unique regions of the genome at several loci simultaneously. The primer is composed of a microsatellite sequence anchored at 3′ or 5′ end by 2–4 arbitrary, often degenerate nucleotides (Qian et al. 2001). Several properties of microsatellites, such as high variability among taxa, ubiquitous occurrence and high copy number in eukaryotic genomes (Weising et al. 1998), make ISSRs extremely useful markers. They exhibit specificity of sequence-tagged-site markers without the requirement of any prior knowledge of genome sequence for primer synthesis (Joshi et al. 2000). ISSR technique has been employed for phylogenetic relationship study in many crops such as rice (Joshi et al. 2000; Qian et al. 2001). ISSR has become a popular technique for genetic relationship study by many scientists working on bamboo (Lin et al. 2010; Mukherjee et al. 2010).

Several studies have been conducted in rice which include work done by Rabbani et al. in the year of 2008. They have employed RAPD analysis for genetic diversity assessment and identification of 10 traditional, 28 improved and 2 Japanese cultivars of Pakistani rice, where 40 genotypes were grouped into 3 main clusters corresponding to aromatic, non- aromatic and japonica group. A number of improved traditional cultivars originating from different sources did not form well-defined groups and interspersed, indicating no association between the RAPD patterns and geographic origin of the cultivars. Another study was conducted by Qian et al. (2001) for assessment of genetic variation within and among the population of *Oryza granulata* from China using 20 RAPD primers and 12 ISSR primers. Their study showed that RAPD markers revealed a high degree (73.85 %) of genetic variation between the populations residing in two regions; whereas genetic diversity between populations within the same regions was recorded in a very low level. The ISSR primers showed great amount of variation (49.26 %) between two regions coupled with a low level of variation within population and between populations within region. Ten RAPD primers and ten ISSR primers were utilized for detecting DNA polymorphism, identification and genetic diversity study in 16 barley cultivars (Fernandez et al. 2002). One RAPD primer and four ISSR primers were able to distinguish all the cultivars and a strong and quite linear relationship was obtained between resolving power (Rp) of a primer and its ability to discriminate genotypes. RAPD analysis was employed for detection of genetic diversity between *Coffee* species and between *Coffea arabica* genotypes (Orozco-Castillo et al. 1994). The dendrograms were consistent with the known history and evolution of the *C. arabica*. Materials originating from Ethiopia and Arabica sub-groups *C. arabica* var. *typica* and *C. arabica* var. *bourbon* were clearly distinguished. RAPD analysis therefore reflects morphological differences between the sub-groups and the geographical origin of the coffee material.

RAPD analysis was used to estimate genetic diversity and taxonomic relationships in 38 clones belonging to three tea varieties (Wachira et al. 1995). Extensive genetic variability was detected between species, which was partitioned into ‘between’ and ‘within’ population components. RAPD analysis was able to discriminate all of the 38 commercial clones, even those which cannot be distinguished on the basis of morphological and phenotypic traits.

RAPD marker was utilized by Shiran et al. (2007) for detection of genetic diversity of Iranian almond cultivar and their relationship to important foreign cultivars and their relatives. RAPD proved to be more efficient in discriminating genotypes than the SSR markers for the same set of genotypes. For genetic diversity assessment of 20 accessions of sesame (*Sesamum indicum* L.), the RAPD technology was employed by Akbar et al. (2011). RAPD technique revealed a high level of genetic variation among the sesame accessions collected from diverse ecologies of Pakistan. This high level of genetic diversity among the genotypes recommended that RAPD technique is valuable for taxonomic classification of sesame and can be helpful for the upholding of germplasm banks and the competent choice of parents in breeding programs. Very recently Singh et al. (2012), for evaluation of genetic diversity of 60 accessions of turmeric (*Curcuma longa*) from 10 different agroclimatic zones, utilized both RAPD and ISSR primers. Using both RAPD and ISSR markers for 60 genotypes, 62 % correlation between genetic similarity and geographical location were demonstrated. The highest genetic diversity was observed in western central table land.

Many researchers have employed RAPD and ISSR for genetic diversity analysis and identification of bamboo. Nayak et al. (2003) utilized thirty decamer random primers on 12 bamboo species for their identification and genetic relationship study. Selected primers were used for identification and for establishing a profiling system to estimate genetic diversity. Cluster analysis revealed two main clusters which are again divided into three mini clusters. Das et al. (2005) developed two species-specific SCAR markers. Their work involved thirty random decamer primers which were initially screened to detect species- specific markers. Two species-specific RAPD marker Bb_836_ for *B. balcooa* was derived from PW-02 and Bt_609_ for *B. tulda* was derived from OPA-08. Bhattacharya et al. (2006) employed RAPD technology for characterization of *B. tulda*, a gregarious flowering bamboo. The study was conducted based on 32 key morphological characters and 30 random decamer primers (RAPD primers). The molecular clustering pattern is in agreement with classification given by Gamble (1896), while the dendrogram generated from morphological characters differ greatly from it. Phylogenetic relationship among 15 bamboo species was evaluated using morphological and molecular markers (Das et al. 2007). The molecular technique involved RAPD markers, and the dendrogram pattern generated, is in conformity with classical taxonomy. Ramanayake et al. (2007) investigated nine species of bamboo, four (genera) of which are from Sri Lanka, using 41 RAPD primers. Among the four *Bambusa* species, the genetic distances between *B. bambos, B. ventricosa* and *B. vulgaris* were smaller, while *B. atra* differed from them for having greater distance. Smaller genetic distance between *G. atroviolacea* and three *Bambusa* species indicates that *G. atroviolacea* has closure affinity with these three species than *B. atra*. *A. hindsii* which shows greatest distance from all others. In the year 2009, Bhattacharya et al. utilized random decamer primers for molecular characterization of a gregarious flowering bamboo, *T. spathiflorus* subsp. *spathiflorus.* DNA fingerprinting using RAPD markers could not detect any polymorphism either ‘between populations’ or ‘within populations’.

Genetic diversity among twelve accessions of *M. baccifera* from Mizoram was evaluated using RAPD and ISSR markers by Lalhruaitluanga and Prasad (2009). Cluster analysis using Dice similarity coefficient by RAPD markers showed two groups. Similar clustering was found, using Dice similarity coefficient, by ISSR markers. ISSR marker was used for genetic diversity study among 10 cultivars of *P. pubescens* (Lin et al. 2010) where 16 ISSR primers were able to distinguish ten cultivars of *P. pubescens.* Genetic distance and cluster analysis showed that genetic similarity existed between all the cultivars of *P. pubescens* under study. In the year 2010, Mukherjee et al. employed 12 ISSR primers and four EST-based random primers for genetic relationship evaluation among 22 taxa of bamboo. The grouping of genotypes based on Jaccard’s similarity matrix using Unweighted Pair Group Method Arithmetic Average, and Principle Coordinate Analysis agreed with earlier reported molecular phylogenetic study only with a few deviations.

Lin et al. (2010) performed crossbreeding of two *Phyllostachys* species and for the identification of their hybrid they utilized eight ISSR primers. Using ISSR markers, they identified three hybrids produced by the cross. The fingerprinting pattern and genetic distance measure suggest that two hybrids were authentic, whereas the third one probably an intraspecies offspring. Genetic diversity among 12 natural populations of *D. membranaceus* was assessed as a preliminary analysis for protection of germplasm resources using ISSR markers (Yang et al. 2012). They have reported a large proportion of genetic variation among the members ‘within populations’, while the lower genetic variation was found ‘among populations’. No significant correlation between genetic and geographic distances ‘among populations’ was found.

## Abbreviation

AFLP: Amplified fragment length polymorphism
ISSR: Inter simple sequence repeat
RAPD: Randomly amplified polymorphic DNA
RFLP: Restriction fragment length polymorphism
SNP: Single nucleotide polymorphism
SSR: Simple sequence repeat
